# On love: *Variations on Dante & Cassidy*

**DOI:** 10.1017/S147895152610323X

**Published:** 2026-07-23

**Authors:** Miguel Julião

**Affiliations:** Cuidados Paliativos, Equipa Comunitária de Cuidados Paliativos PalCo, ULS Amadora/Sintra, Portugal

While watching you go away,
I lay down and wept, not prayed,
for I believe in no gods anymore
but ourselves.

*E pensando di lei*
*Mi sopragiunse un soave sonno*

Thinking of you,
a gentle sleep overtook me,
and with it a nightmare grabbed me,
filled with memories,
that wound as they are only memories,
the ghost of your perfume,
I cannot grasp.

*Ego dominus tuus*
Am I?

*Vide cor tuum*
“Look upon your heart,”
you say, even as you leave.

*E d’esto cor ardendo*
In the hand of Love,
my heart it burns,
the sound of sparks in the air …

*Cor tuum*
*Umilmente pascea*
yes, my heart …
broken, shattered,
devoured from within.

*Appresso gir lo ne vedea piangendo*
I weep.
I descend into the deepest chamber
of my sorrow.
Without you,
I remain where you left me.
My body becomes the earth
beneath its own weight.

*La letizia si convertia in amarissimo pianto*
Joy turns
into the bitterest tears.

*Io sono in pace*

Are you? …

*Cor meum*
My heart,
my fallen, burning heart.

*Io sono in pace*

I know you are …

*Vide cor meum*
Please turn back
and look upon my heart once more
as your silhouette fades.

*__*

*Historia nostra*
*in pectore manet*
